# Orexin, serotonin, and energy balance

**DOI:** 10.1002/wsbm.1536

**Published:** 2021-09-15

**Authors:** Vijayakumar Mavanji, Brianna Pomonis, Catherine M. Kotz

**Affiliations:** ^1^ Research Service Minneapolis VA Health Care System Minneapolis Minnesota USA; ^2^ Department of Integrative Biology and Physiology University of Minnesota Minneapolis Minnesota USA; ^3^ Geriatric Research Education and Clinical Center Minneapolis VA Health Care System Minneapolis Minnesota USA

**Keywords:** energy expenditure, orexin, physical activity, serotonin

## Abstract

The lateral hypothalamus is critical for the control of ingestive behavior and spontaneous physical activity (SPA), as lesion or stimulation of this region alters these behaviors. Evidence points to lateral hypothalamic orexin neurons as modulators of feeding and SPA. These neurons affect a broad range of systems, and project to multiple brain regions such as the dorsal raphe nucleus, which contains serotoninergic neurons (DRN) important to energy homeostasis. Physical activity is comprised of intentional exercise and SPA. These are opposite ends of a continuum of physical activity intensity and structure. Non‐goal‐oriented behaviors, such as fidgeting, standing, and ambulating, constitute SPA in humans, and reflect a propensity for activity separate from intentional activity, such as high‐intensity voluntary exercise. In animals, SPA is activity not influenced by rewards such as food or a running wheel. Spontaneous physical activity in humans and animals burns calories and could theoretically be manipulated pharmacologically to expend calories and protect against obesity. The DRN neurons receive orexin inputs, and project heavily onto cortical and subcortical areas involved in movement, feeding and energy expenditure (EE). This review discusses the function of hypothalamic orexin in energy‐homeostasis, the interaction with DRN serotonin neurons, and the role of this orexin‐serotonin axis in regulating food intake, SPA, and EE. In addition, we discuss possible brain areas involved in orexin–serotonin cross‐talk; the role of serotonin receptors, transporters and uptake‐inhibitors in the pathogenesis and treatment of obesity; animal models of obesity with impaired serotonin‐function; single‐nucleotide polymorphisms in the serotonin system and obesity; and future directions in the orexin–serotonin field.

This article is categorized under:

Metabolic Diseases > Molecular and Cellular Physiology

## Graphical abstract

The lateral hypothalamus (LH) orexin (OX) neurons send excitatory projections to the serotonergic dorsal raphe nucleus (DRN). In turn, dorsal raphe neurons project back to the energy homeostasis‐regulating hypothalamus, and to motor control areas such as basal ganglia, motor cortex, cerebellum, and spinal cord. Thus, the LH‐DRN OX projections, and projections from DRN to other motor control areas may be part of a neuromodulatory system that influences spontaneous physical activity and energy expenditure.

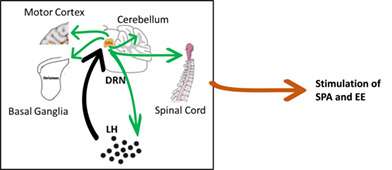

## 1 INTRODUCTION

Neural sensing of body energy status and consequent alteration of behavioral drives such as sleep, appetite, and activity are essential for survival. The hypothalamus is a highly conserved brain area, playing a critical role in maintaining body homeostasis (Bonnavion et al., 2016), including energy balance. Pioneering studies in rats demonstrated that region‐specific lesions to the hypothalamus resulted in alterations in food intake, either inducing overeating and obesity or reductions in food intake, eventually resulting in death (van Galen et al., 2021). Various neuronal populations of the hypothalamus, with several neurotransmitters, neuromodulators, and humoral factors, convey appropriate outputs to downstream extrahypothalamic structures to maintain behavioral and physiological homeostasis. Thus, normal hypothalamic function depends on synchronized output of highly differentiated networks of hypothalamic nuclei that sense homeostatic signals and integrate these signals originating from central and peripheral sources, including body energy status (Gautron et al., 2015; Mavanji et al., 2021; Saper et al., 2005; Saper et al., 2010; Sternson et al., 2013; Williams et al., 2008). Particularly, the lateral hypothalamus is demonstrated to be critical for the control of vigilance states, ingestive behavior, motivated activities, and thermogenesis (Bonnavion et al., 2016). By acting as a focal point, the LH is known to integrate multiple metabolic signals and coordinate behavioral outputs appropriate to the situation and energy needs. Central to this multifunctional influence on physiological processes is the presence of heterogeneous neural populations defined by well‐characterized neurochemical markers and neuropeptides such as hypocretin/orexin. Despite a vast number of studies available on LH functions, defining specific circuitry driving complex behavior remains a significant challenge in the field. The orexin neurons participate in feeding, reward processing, the sleep wake cycle, spontaneous physical activity (SPA), muscle tone, motor activity, energy expenditure and other physiological processes, and act as a focal point for the convergence of information important for these processes (Teske et al., 2010).

## 2 OREXINS

### 2.1 Orexin neuropeptides and their receptors

The orexin system consists of orexin‐A and orexin‐B peptides, along with orexin receptors 1 and 2 (OX1R, OX2R) (de Lecea et al., 1998; Sakurai et al., 1998). Orexins are produced exclusively in the lateral/perifornical and dorsomedial regions of the hypothalamus (Teske & Mavanji, 2012) and project ubiquitously throughout the brain and spinal cord. In addition, orexin is detected in several peripheral tissues (Teske & Mavanji, 2012). Like orexin A and B peptides, OX1R and OX2R are observed throughout the central neuraxis and in discrete peripheral regions (Cluderay et al., 2002; Hervieu et al., 2001; Johren et al., 2002; Trivedi et al., 1998). Interestingly, both OX1R and OX2R are observed in human adipose tissue, and treatment with orexin enhances peroxisome proliferator‐activated receptor gamma (PPAR‐γ) expression, along with enhancing browning of adipose tissue. In addition, orexin treatment of adipose tissue results in increased release of glycerol, indicating higher lipolysis (Digby et al., 2006). These studies indicate that targeting adipose tissue with orexin may be a potential therapy for metabolic dysfunctions such as obesity and hyperglycemia (Digby et al., 2006). Even though OX1R and OX2R are concurrently present, they are unevenly distributed within the brain and spinal cord, suggesting that they may play differential physiological roles (Brown et al., 2002; Cluderay et al., 2002; Digby et al., 2006; Hervieu et al., 2001; Mavanji et al., 2010; Ohno & Sakurai, 2008; Teske & Mavanji, 2012; Trivedi et al., 1998). Affinity of orexin A is equal for both OXR's, whereas orexin B's affinity is five times greater for OX2R compared with that for OX1R (Sakurai et al., 1998). Orexins have excitatory as well as inhibitory postsynaptic effects (de Lecea et al., 1998). The orexin field projects to several brain areas implicated in physical activity, including the locus coeruleus, dorsal raphe nucleus (DRN), and substantia nigra (C. Kotz et al., 2012). Earlier studies indicate that orexin receptors (OXRs) have varying affinities for OXA and OXB, and orexin receptor binding activates several G‐proteins, such as Gq, Gs, and Gi/o, indicating complex intracellular orexin signaling cascades (Sakurai et al., 1998; L. Zhang et al., 2009).

The differential affinity of orexins for its receptors may influence the effects of orexins on physiological functions. For example, narcoleptic mice with underlying orexin deficiency are prone to weight gain, and rats with higher orexin sensitivity do not gain weight when fed a high‐fat diet (Kakizaki et al., 2019; Teske et al., 2006). Yet, it is difficult to parse out the role of individual OXRs in susceptibility to weight gain. Numerous studies suggest that the stimulation of OX1 and OX2 receptors results in independent functional outcomes. For example, earlier studies showed that the sleep disorder canine narcolepsy is caused by a mutation in the OX2R gene (Sakurai, 2014). In addition, it has been demonstrated that OX2R KO mice and double orexin‐receptor KO mice exhibit a narcoleptic phenotype, whereas OX1R KO mice showed only a mild fragmentation of sleep/wake states (Sakurai, 2014; D. Zhang et al., 2021). Moreover, while the effects of orexin‐A on wake promotion and sleep suppression were attenuated in both OX2R and OX1R KO mice, substantially greater reductions were observed in OX2R KO mice, indicating that sleep appears to be primarily regulated by OX2R and to a lesser extent by OX1R (D. Zhang et al., 2021). In contrast, food intake, emotion, autonomic regulation, and reward‐related behaviors are shown to be most closely tied to OX1R receptors (Sakurai, 2014).

Several studies have demonstrated the role of OX1R in eating behavior. (Cason & Aston‐Jones, 2013a, 2013b; D. L. Choi et al., 2012). The OX1R antagonist SB334867 attenuates home‐cage and OXA‐induced feeding (Haynes et al., 2002; Nair et al., 2008; Sakurai, 2014); high‐fat pellet self‐administration in food restricted rats, and ad‐libitum fed mice (Cason & Aston‐Jones, 2013a, 2013b; Sharf et al., 2010); binge‐like consumption of palatable food in mice (Alcaraz‐Iborra et al., 2014) and rats (Freeman et al., 2021); and cue‐driven food consumption (Cason & Aston‐Jones, 2013a, 2013b; Cole et al., 2020; Kay et al., 2014). Similarly, knockdown of OX1R in paraventricular nucleus of the thalamus (PVT) using OX1R shRNA reduces palatable food intake in mice (D. L. Choi et al., 2012), and high‐fat food‐conditioned place preference was inhibited by fourth ventricular administration of SB334867 (Kay et al., 2014). A rat study demonstrated that OX1R signaling mediates the consolidation and recall of Pavlovian cue–food association, as well as its extinction (Keefer et al., 2016), and oral GSK1059865, a selective OX1R antagonist, and i.p. SB33487 reduced binge eating in mice and female rats, respectively (Alcaraz‐Iborra et al., 2014; Piccoli et al., 2012).

A recent study showed that lack of OX1R results in obesity resistance in mice given a high‐fat diet, whereas mice lacking OX2R showed lower thermogenesis when receiving a high‐fat diet. Moreover, mice deficient in either OX1R or OX2R gained weight equivalent to that observed in narcoleptic mice, that lack orexin (Kakizaki et al., 2019). These results may appear to be contradictory, but demonstrate the concept that signaling of each orexin receptor may uniquely influence energy balance. Importantly, results from studies in gene knock‐out animal models need to be interpreted with caution, as compensatory responses at the intact OX receptor may yield inconclusive outcomes. The study by Kakizaki et al., also showed suppression of diet‐induced obesity (DIO) in wild type (WT) mice in the presence of a running wheel, an effect which was attenuated in orexin‐deficient mice, indicating that orexin neuron signaling interacts with both diet and exercise in body weight regulation (Kakizaki et al., 2019). In sum, orexin signaling increases not only food intake but also energy expenditure, and an increase in the net orexin tone generally results in thermogenesis and decreased body weight gain (Sakurai, 2014).

### 2.2 Orexin, spontaneous physical activity, and energy expenditure

Inherent biological mechanisms and environment influence susceptibility to obesity in humans and animals (C. M. Kotz et al., 2017). Susceptibility to DIO and co‐morbidity of obesity varies extensively between individuals (Jordan et al., 2012; C. M. Kotz et al., 2017). Variability in obesity susceptibility in response to diet is partially determined by thermogenesis resulting from SPA, referred to as non‐exercise induced thermogenesis (NEAT) (C. M. Kotz et al., 2017). Spontaneous physical activity in humans presents as fidgeting, standing, and ambulating (Garland Jr. et al., 2011; Vanltallie, 2001). It is believed that SPA reflects non‐goal‐oriented activity (such as high intensity voluntary exercise), but emanates from a subconscious drive for movement (C. M. Kotz & Levine, 2005; Levine et al., 2006). Approximately 30% of daily energy expenditure (EE) is attributed to SPA and NEAT in humans (C. M. Kotz et al., 2017), and is a major determinant of individual susceptibility to DIO (Levine et al., 1999). Some individuals increase their SPA and NEAT to resist obesity when overfed, whereas others do not, suggesting that SPA and NEAT variability among individuals critically contribute to energy homeostasis (Levine et al., 1999). Similar to human studies, most animal studies support the idea that SPA and NEAT confer protection against obesity. Mechanisms controlling NEAT and exercise involve orexin neurons (Garland Jr. et al., 2011; Nixon et al., 2012; Teske et al., 2008), and our previous work shows that the orexin system is central for the regulation of vigilance states, SPA, NEAT, and energy balance (C. M. Kotz et al., 2017), and that LH orexin is protective against weight gain (Bunney et al., 2017; DePorter et al., 2017; Levin et al., 1997; Z. Liu et al., 2020; Teske et al., 2006). For instance, earlier studies using rats that were selectively bred as obesity‐prone (OP) or obesity resistant (OR, based on weight gain profiles following exposure to a high fat diet) (Levin et al., 1997), indicated that OR rats show enhanced intrinsic SPA, and orexin‐induced SPA as compared with that of control and OP rats (Teske et al., 2006). In another study, we used high activity (HA) and low activity (LA) rats, where rats were classified based on their intrinsic SPA, into HA or LA rats. This study showed that similar to OR rats, HA rats resist obesity following exposure to high‐energy diet in comparison to that of LA animals, and exhibit higher behavioral sensitivity to orexin (Perez‐Leighton et al., 2012). Thus, both OR and HA rats demonstrate that an individual's propensity for SPA significantly determines resistance to diet‐induced obesity. In a recent study, OR mice (mouse strain phenotypically identical to OR rats) increased their SPA when exposed to a cafeteria diet, without exhibiting any difference in their SPA prior to cafeteria diet exposure (Gac et al., 2015). On the other hand, in obesity susceptible C57 mice, high fat diet (HFD) feeding decreased their SPA and NEAT (Moretto et al., 2017), indicating that increased SPA and NEAT promotes obesity resistance. Moreover, physical exercise increases plasma orexin A levels, which activates the sympathetic nervous system and energy expenditure (C. M. Kotz et al., 2017; Monda et al., 2020; Polito et al., 2020). Further, exercise increases the activation of orexin neurons (James et al., 2014), and dual orexin receptor antagonist (DORA) oral administration decreases SPA for 8 h and core body temperature (CBT) for 4 h, with the CBT response being independent of SPA. Similarly, exercise‐induced enhancement of CBT via treadmill running was blunted after DORA administration, further supporting a role of orexin in thermoregulation during exercise (Martin et al., 2019). Thus, it is possible that a secondary increase in OXA during exercise promotes thermogenesis.

Activity of orexin neurons is entrained to waking, as well as to external and internal signals such as fasting and caloric restriction (Alam et al., 2005) indicating their prominent role in sleep/wake and energy homeostasis regulation. Orexin neuron activity is influenced by metabolic state indicators and intra‐hypothalamic and extra‐hypothalamic inputs (glucose, leptin, and amino acids), such that their firing is higher during the wake state and fasting (Gac et al., 2015; Moretto et al., 2017; Sadowska et al., 2017a, 2017b). In addition, orexin neurons are sensitive to ATP and lactate levels, and thus act as energy sensors (Z. W. Liu et al., 2011; Parsons & Hirasawa, 2010). Orexin neurons influence homeostatic and physiological behaviors such as food intake, attention, sleep/wake cycle, locomotion, addiction, learning, and memory through widespread neuronal projections to regions that mediate these phenomena (C. M. Kotz et al., 2017; Mavanji et al., 2015; Stanojlovic, Pallais, & Kotz, 2019, Stanojlovic et al., 2021; Stanojlovic, Pallais, Lee, & Kotz, 2019; Stanojlovic, Pallais Yllescas Jr., Mavanji, & Kotz, 2019). Importantly, orexin neurons increase SPA in both sexes (Bunney et al., 2017; Zink et al., 2018), which is mediated by several brain sites (Kiwaki et al., 2004; C. M. Kotz et al., 2006; Teske et al., 2013; Thorpe et al., 2006; Thorpe & Kotz, 2005) and GABAergic neurons within the LH (C. M. Kotz et al., 2006). Orexins have also been shown to enhance eating behavior, but this is a short‐term effect that is quickly compensated by reduced intake, resulting in no overall long‐term changes in food intake following orexin stimulation (Kiwaki et al., 2004; C. M. Kotz et al., 2002; Nishino et al., 2001). In addition, central injection of orexin or brain orexin overexpression decreases body weight gain in animals (Funato et al., 2009; Novak & Levine, 2009; Perez‐Leighton et al., 2012). Obesity resistant rats have greater orexin receptor gene expression and orexin behavioral sensitivity (Perez‐Leighton et al., 2012; Teske et al., 2006; Teske et al., 2008); are more physically active; and have consolidated sleep (Mavanji et al., 2010; Teske et al., 2006), indicating a protective effect of orexin against obesity. As detailed above, physical activity results in energy expenditure and orexin consistently promotes SPA (Teske & Mavanji, 2012). Physically active individuals exhibit higher plasma levels of orexin (Hao et al., 2017). Further supporting the role of orexin in obesity resistance, it has been shown that orexin A injected into the rostral LH and paraventricular nucleus (Kiwaki et al., 2004; Novak et al., 2006) increases NEAT (C. M. Kotz et al., 2017; C. M. Kotz et al., 2002; C. M. Kotz et al., 2006; Teske et al., 2010), and repeated LH orexin injections injection reduces adiposity (Perez‐Leighton et al., 2013). In addition, orexin injected into tuberomammillary nucleus, medial preoptic area, DRN, nucleus accumbens, substantia nigra, ventrolateral preoptic area (VLPO), and locus coeruleus (España et al., 2001; Kiwaki et al., 2004; C. M. Kotz, 2006; C. M. Kotz et al., 2008; C. M. Kotz et al., 2002; Mavanji et al., 2015; Novak et al., 2006; Novak & Levine, 2009; Teske et al., 2010; Teske et al., 2006; Teske et al., 2013; Thorpe & Kotz, 2005) enhances SPA and EE. Moreover, micro‐infusion of orexin into several brain regions increased EMG activity and muscle tone (Kiyashchenko et al., 2002; Mileykovskiy et al., 2002, 2005; Peever et al., 2003; Teske & Mavanji, 2012). In a recent study, we showed that optogenetic stimulation of orexin neurons increases SPA (C. M. Kotz et al., 2017). Using designer receptors exclusively activated by designer drugs (DREADD), we showed that chemogenetic orexin neuron activation increases SPA and NEAT, and prevents obesity in mice given a high‐fat diet (C. M. Kotz et al., 2017; Zink et al., 2018). Moreover, DREADD‐induced activation of the orexin neuronal field mitigated aging‐related reductions in SPA and EE (Stanojlovic, Pallais Yllescas Jr., Mavanji, & Kotz, 2019). Conversely, orexin antagonists reduce SPA and NEAT (Martin et al., 2019; Mavanji et al., 2015). Similarly, humans with obesity and animal models of obesity exhibit lower physical activity, reduced sleep quality, and orexin levels (in hypothalamus and plasma), whereas enhanced sleep quality and plasma orexin levels are observed following weight loss (Teske & Mavanji, 2012; C. Kotz et al., 2012). A recent study showed that the orexin neurons are necessary for movement initiation, as optogenetic‐silencing of these cells reduced movement initiation, without affecting ongoing movement (Karnani et al., 2020). In addition, orexin is largely sympathoexcitatory (Teske & Mavanji, 2012). Studies in rodents demonstrate that orexin enhanced sympathetic outflow as indicated by elevated blood pressure and heart rate (Teske & Mavanji, 2012), increased renal sympathetic nerve activity including to the brown adipose tissue (BAT) (Straat et al., 2020), and elevated plasma epinephrine, noradrenaline release, and firing rate of sympathetic nerves (Teske & Mavanji, 2012). This indicates that orexin‐induced NEAT has “extra” caloric expenditure which may be via sympathetic output to BAT (Morrison et al., 2014). Thus, orexins are important for energy balance, via their afferent projections throughout the CNS, including to areas crucial for the regulation of physical activity, such as the DRN, locus coeruleus, and substantia nigra (C. Kotz et al., 2012).

**FIGURE 1:**
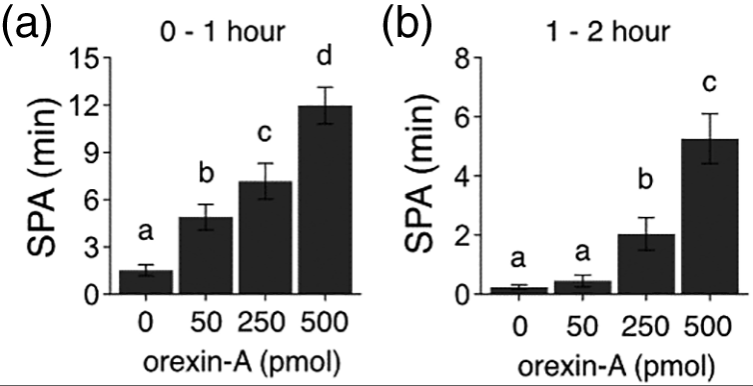
DRN‐injected orexin increases SPA. Plots A and B represent the first and second hour post‐injection time intervals. Two‐way repeated measures ANOVA (dose as repeated) indicates significant effects of different doses of orexin A at 1 and 2 h post‐injection. Post‐hoc testing by Holmes comparison indicates that all doses are significantly different from baseline at 1 h, and the 250 and 500 pmol dose are significantly greater at 1–2 h post‐injection. Bars with differing superscripts indicate that the representative means are significantly different from each other, *p* < 0.05; *N* = 11

Orexin‐deficient humans with narcolepsy, and animal models of orexin loss show a propensity for weight gain, despite decreased caloric intake (Hara et al., 2001). Over‐expression of orexin results in obesity resistance in mice (Funato et al., 2009), and existing studies suggest that obesity resistance in animal models is associated with; (a) higher behavioral sensitivity to orexin A (Teske et al., 2006), (b) greater LH prepro‐orexin expression, and (c) higher rostral LH orexin A sensitivity to enhance SPA (Perez‐Leighton et al., 2012). As described above, OXR subtypes in many brain regions are involved in orexin‐dependent regulation of energy expenditure (C. M. Kotz et al., 2017), including the rostral LH (C. M. Kotz et al., 2017), ventral lateral preoptic area, DRN, substantia nigra, and locus coeruleus (Hao et al., 2017; C. Kotz et al., 2012; C. M. Kotz et al., 2017) (Figure 1).

## 3 OREXIN–SEROTONIN INTERACTIONS

### 3.1 Orexin–serotonin interaction, physical activity, and energy expenditure

As described above, orexin acts via multiple sites to promote SPA energy expenditure. We have new data showing that DRN may be one such important target for orexin stimulation of SPA and energy expenditure (Figure 1). The DRN neurons of the brainstem are a key site for serotonin/5‐hydroxytryptophan production in the CNS (Xiao et al., 2017), and are important for regulating mood, behavior, appetite, and thermogenesis. Serotonin is the primary neurotransmitter in DRN and thus represents a likely candidate for the SPA‐ and NEAT‐ inducing effects of orexin action in DRN (W. Choi et al., 2020). Apart from its role in motor function, central serotonin acts as an anorectic agent, even though the opposite function is attributed to peripheral serotonin systems (Kesić et al., 2020; Yabut et al., 2019). Dysfunction of central serotonin signaling results in increased food intake and weight gain in humans and animals (Yabut et al., 2019), whereas serotonin agonists decrease food intake (Yabut et al., 2019). The majority of serotonergic outputs important for energy balance project mainly from the DRN and the median raphe nucleus (MRN) (van Galen et al., 2021), and elevating brain 5‐HT function is suggested as a potential antiobesity therapy (Burke & Heisler, 2015).

Excitatory orexin terminals are found in the DRN, and both orexin receptors are located on serotonin cell bodies within the DRN, a structure which is also an important part of the ascending arousal system (Darwinkel et al., 2014; Mavanji et al., 2010; Yaswen et al., 1999). It has been demonstrated that OX2R mediates orexin influence in the DRN, and OX2R dysfunction results in narcolepsy (Lin et al., 1999; R. J. Liu et al., 2002). Conversely, serotonergic neurons from raphe nuclei project to the lateral hypothalamic orexin neurons, where serotonin inhibits orexinergic cells (Muraki et al., 2004; Yamanaka et al., 2003). Moreover, serotonergic neurotransmission in the hypothalamus is a component of the energy homeostasis network which receives satiety‐related signals from the periphery to modulate appropriate central responses (Heisler et al., 2006). The 5‐HT1A, 5‐HT2C, and 5‐HT1B serotonin receptors are localized on orexin neurons, and blunted serotonin transmission in lateral hypothalamus and perifornical area is associated with obesity, altered feeding, and sleep/wake cycles underscoring the complex role of hypothalamic serotonin in energy balance (Romanova et al., 2018).

Serotonin release occurs in dorsal, but not medial, raphe nuclei after orexin administration into these sites (Tao et al., 2006), and changes in monoamines in the DRN affect behavior (Weiss et al., 2008). Our earlier study has shown that OXR1 and OXR2 mRNA are present in the DRN, and these mRNA are elevated in obesity resistant (OR) rats (Mavanji et al., 2010). Recently, we found that, orexin A in DRN increased SPA without affecting food intake (Figure 1). In addition, serotonin antidepressant drugs are widely used among those with obesity and chronic injuries, suggesting its role in energy balance and NEAT, and one such drug, fluoxetine, increases the expression of OX2R in the thalamus and hypothalamus (Bross & Hoffer, 1995; Nollet et al., 2011). Studies have shown that the OXR expression pattern, non‐resting thermogenesis, and behavioral (SPA) sensitivity to orexin A as well as orexin receptor distribution in the DRN differentiate OR rats from control rats, identifying the DRN as a structure influencing orexin A‐induced enhancement of SPA (Mavanji et al., 2010; Teske et al., 2013). Even though peripheral serotonin is part of energy conservation (W. Choi et al., 2020; Kesić et al., 2020; Yabut et al., 2019), the above studies point to an important role of DRN serotonin in energy expenditure effects of orexin A. Even in primitive organisms such as *Caenorhabditis elegans*, central serotonin increases energy expenditure and decreases body fat by enhancing oxidation of fat (Yabut et al., 2019). Moreover, fluoxetine (FLX), a selective serotonin reuptake inhibitor (SSRI), reduces weight gain by decreasing fat mass, feeding behavior, and by increasing the expression of hypothalamic neuropeptides involved in satiety (da Silva et al., 2019). In addition, FLX administration enhanced energy efficiency, mitochondrial respiratory chain function, redox state, and mitochondrial biogenesis in neonatally overfed rats, which might be an adaptive response to nutritional imbalance in early life, further supporting a role for central serotonin in energy expenditure (Braz et al., 2020). Apart from directly modulating motor activity and sympathetic outflow to the BAT as described in later sections, serotonin–orexin interactions might occur in other brain areas to modulate energy homeostasis.

### 3.2 Serotonin and brown adipose tissue thermogenesis

As indicated above, central serotonin is also involved in promoting thermogenesis by regulating BAT thermogenesis, and glucose and fat homeostasis (McGlashon et al., 2015). During exposure to cold, serotonin neurons in the raphe nucleus increase their firing and BAT thermogenesis, whereas blocking serotonin receptors affects cold‐exposure related BAT activity (McGlashon et al., 2015). In addition, central serotonin is required for inducing uncoupling protein 1 (UCP1) expression and browning of white adipose tissue (WAT) (McGlashon et al., 2015). In the McGlashon et al., study, inhibition of central serotonin signaling resulted in lower BAT temperature compared with control mice, and resulted in lower BAT activity as indicated by a larger size of lipid droplets on BAT tissue under mild cold conditions. This study suggests that central serotonin is essential for mediating the temperature‐dependent activity and thermogenesis in BAT. Further supporting the role of serotonin in thermogenesis and energy homeostasis, serotonin deficient mice had higher levels of plasma glucose, fatty acids, triglycerides, corticosterone, and thyroid stimulating hormone (TSH) in mild cold condition, an effect which was eliminated when animals were in thermoneutral housing. Serotonin enhances thermogenesis by increasing sympathetic output to BAT (Yabut et al., 2019), and serotonin deficient mice exhibit significant reductions in BAT activity markers, without a change in BAT differentiation markers. Moreover, chronic serotonin reuptake inhibition in postnatally overfed young‐adult rats results in uncoupling of BAT mitochondria, beiging/browning of WAT and reduces feeding behavior and body weight (Braz et al., 2020; da Silva et al., 2019). A recent study suggests that the energy expenditure promoting effects of serotonin may be partly attributed to activation of the 5‐HTR1A and 5‐HTR7 receptors within the spinal cord (Faure et al., 2006), and blockade of central serotonin signaling leads to deficient thermoregulation (Yabut et al., 2019). This loss of thermoregulation results from a decrease in UCP1 levels in both brown fat and WAT (Yabut et al., 2019). A similar phenomenon (deficient BAT and beige fat depots) also occurred in serotonin deficient Lmx1bf/fePet1Cre mice (McGlashon et al., 2015). The complexity of serotonin regulation of energy expenditure is highlighted by the fact that despite the stimulation of thermogenesis by serotonin, mice deficient in central serotonin (due to reductions in the serotonin synthesizing enzyme tryptophan hydroxylase 2 [Tph2]) exhibit a lower body weight and higher thermogenesis (Yabut et al., 2019). Supporting the role of the orexin–serotonin axis in thermogenesis, exogenously administered orexin stimulates sympathetic outflow and direct injection of orexin into the DRN neurons also increases sympathetic stimulation of BAT (Morrison et al., 2012; Morrison et al., 2014; Tupone et al., 2011). In addition, mice lacking orexin have impaired BAT thermogenesis (Sellayah et al., 2011; Vijgen et al., 2011). These studies demonstrate that orexin increases thermogenesis, potentially via stimulating sympathetic outflow and inhibiting parasympathetic output, partly by interaction with DRN serotonin (Kornum et al., 2011; Mochizuki et al., 2006; Teske & Mavanji, 2012). Given the role of orexin in energy balance, and the direct orexin projections onto DRN serotonin neurons, there is neuroanatomical and functional evidence that serotonin's involvement in energy balance is modulated by orexin. In the following sections, we briefly discuss the role of serotonin in motor function, SPA, and the effect of manipulation of serotonin transporters, serotonin receptors, and serotonin reuptake on energy balance, as well as animal models of serotonin deficiency. Finally, we will summarize studies that link single‐nucleotide polymorphisms in serotonergic genes to obesity.

### 3.3 Serotonin, awake state and movement control

Initial evidence for the role of serotonin in movement control came from studies showing reduced serotonin levels in the basal ganglia and cerebellum of movement disorder patients (Kawashima, 2018). Neural projections from and to DRN are identified from basal ganglia and other motor control areas such as the motor cortex, cerebellum, and spinal cord (Kawashima, 2018), and serotonergic agents have been used to treat Parkinson's disease (Kawashima, 2018). Moreover, neural activity of serotonergic DRNs correlates with muscle tension and intensity of locomotor activity as observed during treadmill activity in cats (Kawashima, 2018). Earlier studies showed that serotonin excites spinal cord motor neurons, and enhances the frequency of fictive locomotor‐activity (Kawashima, 2018). In another study, depression‐like behavior was observed in mice, following DRN GABA administration (Xiao et al., 2017). In addition, it has been shown that serotonergic neurons in the DRN encode multiple behavioral variables, such as sensory input, motor action, and reward in rodents (Ranade & Mainen, 2009), indicating that DRN serotonin is important for motor function. Background cortical activation and desynchronization during arousal is necessary to perform purposeful motor behavior. Serotonin orchestrates with other neuromodulators to cause cortical desynchronization during the awake state (Dringenberg & Vanderwolf, 1998), and dysfunction of the serotonin system leads to a loss of behavioral control (Brown et al., 2001; Lucki, 1998; Sasaki‐Adams & Kelley, 2001). Importantly, the forebrain areas primarily receive their serotonin projections from the DRN (Jacobs & Azmitia, 1992), and similar to other wake promoting monoamines, most of the serotonin neurons in the DRN fire tonically during waking, fire considerably less during slow‐wave sleep, and are silent during paradoxical sleep (Jacobs et al., 2002; Sakai & Crochet, 2001; Trulson & Jacobs, 1979) highlighting its role in wake promotion. Orexin A strongly excites DRN serotonin neurons (Brown et al., 2001; Brown et al., 2002), and the DRN exciting property of orexin is mediated by OX2Rs (Brown et al., 2002). In addition, central orexin induced behavioral changes (such as grooming) were attenuated by antagonists of 5‐HT2A and 5‐HT2C receptors (Donovan & Tecott, 2013; Duxon et al., 2001; Matsuzaki et al., 2002), suggesting that serotonin is partly involved in the behavioral effects of orexin. Thus, the orexin–serotonin system is an important part of the vigilance state and motor control system of the brain.

Further supporting the role of orexin–serotonin interactions in promoting motor function, we have shown that orexin stimulation of DRN enhances SPA, without altering food intake in rats (Figure 1). As shown in Figure 1, there is a dose‐dependent and robust increase in SPA, which translates to increased NEAT (thermogenesis due to SPA).

Our published work shows higher OXR expression in several brain areas including the DRN of obesity resistant rats (Mavanji et al., 2010). Accordingly, higher orexin signaling via OXRs in the DR may help maintain optimal energy balance and body weight. These data are exciting because of the physiological nature of the response, and because it is in an orexin projection site that has the potential to be manipulated by serotonergic drugs to promote SPA and energy expenditure.

### 3.4 Serotonin transporter and obesity

A study in humans showed that specific binding ratio of serotonin transporter (SERT) in the pons was positively correlated, with BMI and negatively correlated with BMI in midbrain of obese subjects (Nam et al., 2018). This indicates that increased SERT recruitment in the pons possibly is a result of increased reward processing from increased food intake in obese subjects, resulting in decreased 5‐HT signaling (Nam et al., 2018). Another study showed a positive correlation between methylation of SERT gene with BMI, body weight, and waist circumference in obese subjects (Zhao et al., 2013). A study in rats showed that rats made obese by high carbohydrate diet exhibited significantly higher SERT expression, further supporting the role of serotonin turnover in the development of visceral obesity. This study also showed serotonergic compensatory mechanisms in response to increased carbohydrate consumption as opposed to protein or fat consumption, pointing to a potential role of serotonin in influencing palatability and reward in addition to energy homeostasis (Spadaro et al., 2015). Similarly, another study demonstrated alterations in hypothalamic serotonin turnover in DIO rats (Hassanain & Levin, 2002). In this study, the diet‐resistant (obesity resistant, OR) rats had lower serotonin turnover (prolonging serotonin action) in ARC and PVN at the end of the light cycle, whereas DIO rats did not exhibit this effect until they were obese. In addition, after 48 h of food deprivation, serotonin turnover in the VMN and perifornical hypothalamus (PFH) of DIO rats was markedly decreased, which might be a mechanism to increase food intake following deprivation. This study also suggested diurnal effects of serotonin on energy homeostasis, which may be dependent on the pre‐existing condition of obesity, but likely does not have an influential role in reaching this obese state. Moreover, in this study the anorectic role of serotonin was highlighted by the reduced serotonin turnover at the end of the light cycle, consistent with food foraging behaviors/initiating feeding at this time of day, indicating that serotonin is no longer acting to inhibit appetite during this time‐period. Stimulation of serotonin receptors in the VMN reduces food intake, and VMN serotonin release is lower in obese Zucker rats (Routh et al., 1994). Levels of SERT protein were lower in the infundibular nucleus (equivalent to the ARC in rodents) of postmortem brain tissue of humans who were overweight/obese (Borgers et al., 2014; Manocha & Khan, 2012). In addition, low CSF serotonin and its metabolite levels are observed in obese women when compared with lean women (Strombom et al., 1996). Interestingly, PET or SPECT studies (measuring serotonin receptor or SERT) consistently show reduced serotonin levels and signaling in several brain areas of obese individuals (van Galen et al., 2021). Thalamic SERT levels were higher following consumption of low‐calorie diet for 4‐weeks, where most of the daily intake was consumed as breakfast, highlighting a possible role for serotonin in meal timing. On the other hand, SERT levels were lower, when most of the daily calorie intake occurred during dinner (Versteeg et al., 2017). Moreover, a 6‐week hypercaloric snacking diet, resulted in reduction of diencephalic SERT in lean men (Koopman et al., 2013). Thus, it is possible that overconsumption results in changes to serotonergic signaling during an initial period of over consumption, contributing to the development/continuance of a higher body weight (Koopman et al., 2013; Versteeg et al., 2017).

### 3.5 Serotonin receptors, leptin, and obesity

Absence of leptin receptors on brain‐stem serotonin neurons in mice lead to obesity, and these mice exhibit characteristics identical to obese ob/ob mice (Yadav et al., 2009; Yadav et al., 2011). Serotonin affects neuronal accumulation of leptin, and accordingly, it was suggested that by regulating leptin internalization, and subsequent action of this hormone in the brain, serotonin could affect feeding and energy homeostasis. Supporting this idea, DRN and hypothalamic leptin accumulation is negatively correlated with brain serotonin level in rats (Fernandez‐Galaz et al., 2010).

The activation of 5‐HT2C receptor is suggested to be the main mechanism for the role of serotonin in energy homeostasis. For example, a study using 5‐HT2C receptor mutant mice showed alterations in mRNA expression for proteins that are important to energy balance, hyperphagia, and late‐onset obesity in these mice (Nonogaki et al., 2002). Oxygen consumption was decreased in these animals with an increase in uncoupling protein‐2 (UCP‐2) gene expression in WAT and skeletal muscle. Similarly, UCP‐2 gene expression was increased in the liver of these mutant mice. In addition, the 5‐HT2C receptor mutants exhibited age‐related reductions in beta 3‐adrenergic receptor (beta 3‐AR) gene expression in WAT, indicating that 5‐HT2C receptor dysfunction leads to reductions in beta 3‐AR mRNA levels, resulting in further reductions in thermogenesis and increased adiposity (Nonogaki et al., 2002). A number of studies used a transgenic strategy to increase controlled expression of 5‐HT2C receptor in POMC neurons of mice otherwise lacking 5‐HT2C receptors (Donovan & Tecott, 2013; Y. Xu et al., 2008; Y. Xu et al., 2010). The expression of 5‐HT2C receptor specifically on POMC neurons prevented overconsumption, over‐sensitivity to DIO, insulin resistance, and insensitivity to serotonin agonists (to induce anorexia) in 5‐HT2CR deficient mice (Donovan & Tecott, 2013; Y. Xu et al., 2008; Y. Xu et al., 2010).

Several serotonin receptors, such as 5‐HT1A, 5‐HT1B, 5‐HT2A, and 5‐HT7 are implicated in the regulation of food intake and energy homeostasis. Activation of either 5‐HT1B, or as described above, 5‐HT2C receptors produces hypophagia (Peterlin et al., 2010). As described in earlier sections, serotonergic compounds acting on the above receptors stimulate the anorexigenic POMC neurons, resulting in the release of hypothalamic of α‐melanocyte‐stimulating hormone (MSH) (Halford et al., 2007). In addition, fenfluramine, which acts as agonist at both 5‐HT1B and 5‐HT2C receptors, blocks hyperphagia induced by NPY. Moreover, serotonin receptor agonist administration decreases NPY levels, whereas serotonin receptor antagonists increased NPY levels (Peterlin et al., 2010).

### 3.6 Selective serotonin reuptake inhibitors and antiobesity drugs

Selective serotonin reuptake inhibitors (SSRIs) block SERT action, resulting in sustained serotonin neurotransmission. In humans, SSRIs reduce body weight gain. In a recent study, 20 obese women on a formula diet (3 weeks of a 1.76‐MJ/day formula diet) were administered either placebo or fluoxetine (FLX), and thermogenesis was monitored. This study found that the energy expenditure in FLX‐treated subjects increased significantly within 3 days of commencing treatment. Similarly, the basal body temperature of FLX‐treated subjects increased significantly following treatment. In addition, despite identical energy intakes during a caloric restriction protocol, weight‐loss occurred early in the FLX‐treated subjects relative to that in the control subjects. This study indicates that SSRIs enhance thermogenesis in humans (Bross & Hoffer, 1995). Even though now discontinued, the earlier approved antiobesity drugs such as sibutramine (Meridia) and lorcaserin (Belviq) were demonstrated to have serotonin reuptake inhibitory activity, and serotonin agonist activity (at 5‐HT2C receptor), respectively (Hurren & Berlie, 2011; Oberholzer et al., 2015). In addition, lorcaserin effectively lowers calorie intake, weight gain, and cardiometabolic complications in obese individuals (van Galen et al., 2021). Moreover, serotonergic drugs such as fenfluramine and dexfenfluramine are used as obesity treatments (van Galen et al., 2021), further highlighting the role of serotonin in energy balance.

### 3.7 Serotonin in animal models of obesity

Animal models of obesity have been useful in assessing the role of serotonin in obesity. One study compared the differences in DRN levels of the enzyme tryptophan hydroxylase 2 (TPH2, critical for serotonin synthesis) in the DIO and agouti models of obesity (Mikhailova et al., 2019). Both obesity models exhibited higher body weight, adiposity, glucose, insulin, and leptin blood levels compared with control mice. Interestingly, DIO mice that were fed HFD for 16 weeks showed a reduction in TPH2 expression in DRN (−36%) and (+68%) in the VTA, indicating a serotonin deficiency. On the other hand, agouti mice showed a significant increase of TPH2 expression (+42%) in the VTA, which might be a possible compensatory serotonin response to genetic melanocortin obesity in these mice. Another study showed that eliminating 5‐HT1B receptors in male and female mice results in weight gain compared with wild‐type littermate control animals (Bouwknecht et al., 2001). The authors hypothesized that absence of receptors resulted in increased feeding and obesity. Interestingly, food intake in the KO mice did not vary depending on genotype when accounting for body weight, indicating a possible role for serotonin in energy expenditure. The 5‐HT1B's role in hypophagia is theorized to be located in hypothalamic paraventricular nucleus and parabrachial nucleus of the pons based on local administration studies that showed satiety following serotonin administration into these areas (van Galen et al., 2021). The DRN serotonin neurons are modulated by adrenergic and serotonergic neurotransmission, and an electrophysiological study showed alterations in neural activity of DRN cells following application of the adrenergic agent phenylephrine (PE) and serotonin. Results of this current‐clamp experiment demonstrated that DRN of obese Zucker rats exhibits a greater depolarization and higher firing rate following PE than did DRN neurons from lean Zucker rats, indicating an enhanced adrenergic drive in obese animals (Ohliger‐Frerking et al., 2003). Moreover, obese Zucker rats showed higher VMH serotonin release during food intake episodes. This might be a compensatory mechanism to reduce energy intake and induce satiety, and heightened excitability of the DRN neurons might be responsible for the feeding‐induced enhancement of VMH serotonin release in obese rats (Ohliger‐Frerking et al., 2003). On the other hand, reduced baseline serotonin release is reported in the hypothalamus of DIO animals (Meguid et al., 2000; Routh et al., 1994), and quantitative autoradiography studies showed that obesogenic diet feeding in rats alters 5‐HT1A, 5‐HT1B, and 5‐HT2A receptor binding (Huang et al., 2004). The changes in receptor binding indicates decreased serotonin neuron activity and lower serotonin release following access to an obesogenic diet in rats. Moreover, diet‐related alterations in serotonin signaling occur before obesity development, highlighting that impaired serotonin transmission results in the development of obesity (van Galen et al., 2021). A study in fish (*Cichlasoma dimerus*) showed that daily intraperitoneal injection of fluoxetine (20 μg/g) for a 5‐day period resulted in a marked reduction in feeding behavior, weight gain, and total hepatocyte area observed at the end of the experiment. In addition, a marked decrease in glycogen and lipid content and an increase in protein levels in the liver were observed in fluoxetine‐treated fish. Taken together, these results suggest that fluoxetine produces an anorectic effect in *C. dimerus* (Dorelle et al., 2020), indicating the importance of serotonin in energy homeostasis in a wide variety of species.

### 3.8 Single‐nucleotide polymorphisms and obesity

Existing data show that obesity and metabolic disease also result from single‐nucleotide polymorphisms in genes of the serotonergic system (including serotonin receptor genes) (Walley et al., 2009). For example, an association between higher BMI and mutations of serotonin receptor 2A (5‐HT2A) genes are observed in humans (Li et al., 2014). Comparably, there is an association between 5‐HT2A gene mutation and waist circumference (Halder et al., 2007; Rosmond et al., 2002), and between 5‐HT2A gene mutation and presence of heart disease, stroke, and diabetes (Halder et al., 2007; Kring et al., 2009). Similarly, variants of the serotonin 2C receptor (5‐HTR2C) are implicated in obesity (Kring et al., 2009; McCarthy et al., 2005; Pooley et al., 2004), weight gain (Opgen‐Rhein et al., 2010), and higher BMI (C. Chen et al., 2013). Likewise, the presence of variants of the gene responsible for serotonin synthesis, tryptophan hydroxylase 1 (*Tph1*) is correlated with increased BMI and higher waist circumference (Kwak et al., 2012). Moreover, single‐nucleotide polymorphism of serotonin and tryptophan transporters (*SLC6A4* and *SLC6A14*) correlate with weight gain (Durand et al., 2004; Suviolahti et al., 2003), impairments in fat oxidation and higher BMI (Corpeleijn et al., 2010; Fuemmeler et al., 2008; Yabut et al., 2019). All these genetic studies further indicate an association between weight gain and serotonin synthesis.

## 4 POSSIBLE SITES OF SEROTONIN–OREXIN INTERACTION FOR ENERGY HOMEOSTASIS

### 4.1 Arcuate nucleus, orexin, serotonin, and energy balance

In addition to the LH‐DRN mechanism, another area of interest for the orexin–serotonin interactions for energy balance regulation is the hypothalamic arcuate nucleus. It is believed that the arcuate nucleus (ARC) is the main site of anorectic action of serotonin (Heisler et al., 2006). Among the ARC neuropeptides, agouti‐related peptide (AgRP) and neuropeptide Y (NPY) enhance food intake and reduce thermogenesis (Kesić et al., 2020). On the other hand, ARC cocaine–amphetamine‐regulated transcription (CART), proopiomelanocortin (POMC) cells and α‐melanocyte‐stimulating hormone (α‐MSH) inhibit feeding (Morello et al., 2016). The arcuate POMC neurons co‐express leptin receptors (LepR), and serotonergic 5‐HT1B and 5‐HT2C receptors (Romanova et al., 2018). Serotonin acts as a short‐acting satiety signal in the ARC, and via 5‐HT2C receptor activation, promotes the appetite suppressing action of POMC cells, resulting in reduced feeding and body weight (Morello et al., 2016; Romanova et al., 2018). The anorectic action of serotonin is mediated by the 5‐HT1B receptor on the NPY/AGRP neurons of ARC (Heisler et al., 2006), resulting in their inhibition, which may form an output pathway for the suppression of appetite. The number of 5‐HT2C receptors are increased in POMC‐neurons in DIO conditions, but no changes were seen in 5‐HT1B receptor (Romanova et al., 2018). Similarly, the obese agouti mice showed increases in the number of both serotonergic 5‐HT1B and 5‐HT2C receptors (Romanova et al., 2018), indicating adaptations in serotonin signaling due to obesity development. Further supporting the role of POMC serotonin in energy balance, both DOI and agouti mice showed higher sensitivity to 5‐HT2C receptor‐mediated 5‐HT regulation of these cells, which might be a compensatory mechanism to reduce food intake in obese animals. Similarly, increased 5‐HT1BR expression is suggested to compensate for obesity in agouti mice, by acting as an additive factor in enhancing appetite suppressing effects of 5‐HT2C receptor. The orexin neurons project to POMC cells, which, during periods of inactivity, activate POMC neurons, and promote satiety (Morello et al., 2016). The 5‐HT1B receptors are expressed on AgRP neurons, and the AgRP inhibitory axonal projections to POMC neurons, activation of which results in a decrease in melanocortin receptor (MCR) antagonism and increased release of MCR agonist alpha‐MSH which is anorectic (Heisler et al., 2006). Stimulation of 5‐HT1BR using serotonin agonists reduces release of AgRP and feeding. In addition, activation of 5‐HT1B receptors (may be via GABAergic interneurons) induce release of anorectic alpha‐MSH from POMC neurons. Similarly, administration of d‐fenfluramine (5‐HT agonist) enhances satiety in control mice, however, the agouti mice showed no changes in feeding in response to d‐fenfluramine, indicating the involvement of POMC pathway and melanocortin receptor (MCR) in the satiety inducing actions of ARC serotonin (Heisler et al., 2006). Systemic administration of 5‐HT2CR agonist BVT. X activates melanocortin 4 receptors, and reduces food intake (Lam et al., 2008). Similarly, long‐term BVT. X administration in obese mice, enhances POMC mRNA, decreases weight gain, fat mass and feeding behavior, and the effect of BVT.X is dependent on melanocortin‐4 receptor, indicating a link between serotonin and MCR in regulating energy balance (Lam et al., 2008). Thus, 5‐HT2CR agonism may be more potent in affecting energy expenditure than on feeding in long‐term impact on obesity, as mice ate less for the first 2 days of injections but normalized without compensatory feeding afterwards (Lam et al., 2008). Further supporting the importance of POMC neuron 5‐HT2CR in energy homeostasis, a study showed that deficiency of 5‐HT2CR in the POMC neurons leads to insulin resistance, hyperphagia, hyperinsulinemia, obesity, hyperglycemia, and hyperglucagonemia (Yabut et al., 2019). These mice also lack an anorectic response to serotonin agonists (Berglund et al., 2014). Together, these findings demonstrate that POMC neuron 5‐HT2C receptors are involved in the regulation of energy homeostasis and glycemic control (Berglund et al., 2014). Given that the orexin neurons project to POMC cells, which during periods of inactivity, promote satiety by activation of these POMC neurons (Morello et al., 2016), indicates a possible interaction between orexin and serotonin in the ARC to regulate energy balance. As earlier studies on arcuate nucleus primarily addressed energy intake, future studies are needed to confirm the role of serotonin–orexin mechanisms in this brain area on energy expenditure.

### 4.2 Serotonin, PVN, and VMN

Another area of interest for the orexin‐serotonin interaction in energy balance regulation is the paraventricular nucleus (PVN) of the hypothalamus. Orexin neurons project to the PVN (Wang et al., 2019), and one study showed the role of hypothalamic PVN serotonin in the regulation of food intake patterns and selection of macronutrients (Shor‐Posner et al., 1986). Here, direct PVN administration of serotonin in rats resulted in a significantly smaller, shorter, and slower meal for the first meal, and a longer time delay between the first and second meals post‐injection. In addition, serotonin and norfenfluramine administration resulted in altered meal preference in that rats ate significantly fewer kilocalories from carbohydrate and more from protein. On the other hand, QUIP (a 5‐HT agonist) reduced overall calorie consumption, fat consumption, and increased the percent of carbohydrate and protein consumption. Conversely, administration of CYPRO (a 5‐HT antagonist) increased food intake, and resulted in the largest percentage increase in fat and carbohydrate consumption, indicating that serotonin antagonism in PVN shifts macronutrients toward a dietary intake pattern that is associated with obesity and a Western diet (Shor‐Posner et al., 1986). In another study, 2 weeks intra‐PVN administration of serotonin reduced feeding and weight gain only in lean rats, without affecting the body weight of obese Zucker rats (Fetissov & Meguid, 2010). In addition, VMH serotonin release is altered in genetically obese Zucker rats (Fetissov & Meguid, 2010). The orexin neurons project densely to the PVN of the hypothalamus (de Lecea et al., 1998), and 6 days of daily intra‐PVN orexin treatment induced loss of body weight, without affecting feeding behavior. Furthermore, intra‐PVN orexin enhanced physical activity compared with control treatment, indicating that orexin in PVN promotes physical activity, and hence negative energy balance (Novak & Levine, 2009). Another study showed that intra‐PVN orexin reduced palatable snack intake in mice (Alvarez et al., 2018). Given the SPA‐promoting effect of PVN orexin, and feeding reducing actions of PVN serotonin, it is feasible to suggest that both orexin and serotonin in the PVN act in concert to prevent weight gain. Future studies are needed to study the simultaneous effect of PVN orexin and serotonin on components on total energy expenditure.

The ventromedial nucleus of the hypothalamus (VMN) was the first site to be discovered as a key player in the control of energy homeostasis through regulation of ingestive behavior and energy expenditure (Y. H. Choi et al., 2013). The VMN neurons exclusively produce the steroidogenic factor‐1 (SF‐1) (Y. H. Choi et al., 2013) and also express 5‐HT2C receptors. These VMN SF‐1 neurons receive projections from the DRN serotonin neurons (van Galen et al., 2021), and from orexin neurons in the LH area (Wang et al., 2019). Both OX1R and OX2R are expressed in the VMN of hypothalamus (Trivedi et al., 1998), and an earlier study showed that VMN‐injected OXA does not alter food intake in rats (Dube et al., 1999). In addition, administration of serotonin agonist metachlorophenyl‐piperazine (mCPP) into the VMN reduces food intake (Hikiji et al., 2004). The VMN neurons, similar to DRN serotonin neurons, modulate sympathetic outflow to peripheral tissues including BAT (Y. H. Choi et al., 2013). Genetic deletion of SF‐1 neurons in the VMN results in impaired thermogenesis and energy expenditure (Y. H. Choi et al., 2013). Accordingly, these SF‐1‐containing neurons of the VMN are demonstrated to control energy balance by regulating thermogenesis and glycemic control, rather than feeding behavior (van Galen et al., 2021). Given that VMN injected orexin does not increase feeding, and that these neurons receive DRN projections and express 5‐HT2C receptors, there is basis for the idea that orexin and serotonin in the VMN interact to promote energy expenditure by enhancing sympathetic outflow to BAT and thermogenesis.

### 4.3 Serotonin, nucleus accumbens, and ventral tegmental area

The ventral tegmental area (VTA) receives direct projections from serotonergic neurons of DRN (Nieh et al., 2015) and from the LH, a neural pathway that regulates addictive sugar ingestion (Nieh et al., 2015). In addition, the nucleus accumbens core (NAc) receives direct projections from the DRN and from the LH (Van Bockstaele et al., 1993). Moreover, NAc receives indirect input from LH via the paraventricular nucleus of the thalamus (PVT) (Kelley et al., 2005). On the other hand, the VTA reciprocally projects back to the LH via the PVT, and the NAc also reciprocally projects back to the LH (Kelley et al., 2005; Van Bockstaele et al., 1993; van Galen et al., 2021). Recent studies show that 5‐HT2C receptor activation reduces appetite via dopamine (DA) neurons (P. Xu et al., 2017; Yabut et al., 2019) in the VTA (Valencia‐Torres et al., 2017), and in the nucleus of the solitary tract (D'Agostino et al., 2018). Thus, one of the possible mechanisms for suppression of energy intake by serotonin is the LH–VTA–NAc circuitry. As the above studies primarily focused on energy intake, future studies are needed to test the possible role of NAc/VTA circuitry and orexin–serotonin mechanisms in energy expenditure.

## 5 FUTURE DIRECTIONS

Earlier hypothalamus serotonin work was primarily focused on food intake regulation, and studies related to serotonin's impact on energy expenditure are sparse. More studies are needed to assess the role of serotonin in specific hypothalamic nuclei in energy expenditure regulation. Similarly, a limited number of computer modeling studies are available to simulate the relationship between LH orexin and DRN neurons, and expanding these models with regards to energy balance regulation might be beneficial in understanding the role of this circuitry in the development of obesity. Another relevant future study is live brain imaging during exercise to understand the activation of orexin–serotonin circuitry during physical activity, followed by combined orexin and serotonin intervention (activation or inhibition of specific receptors) in specific brain regions to understand their role in energy expenditure. In addition, orexin and serotonin receptor gene expression studies in obese and obesity‐resistant animal models, and metabolic studies with double mutant mice (specific orexin and serotonin receptor KO) would be beneficial to test whether there is an optimal balance between OXA/serotonin systems to maintain an ideal metabolic state. Similarly, long‐term studies during weight loss intervention to assess changes in the serotonin/orexin neural activation is also warranted. Fundamentally, what the field lacks is selective OX agonists to determine the specific contribution of OXA to energy expenditure and other physiological mechanisms. Identification of selective orexin agonists are underway, and serotonin analogues are available. Future studies with serotonin agents such as fluoxetine and small molecule orexin agonists will help fill the knowledge gap in the field of orexin, serotonin and energy metabolism, which will be of translational significance.

## 6 CONCLUSIONS

Existing literature suggests that orexin is a critical regulator of energy balance, and has potential to be used as a neuromodulator for therapeutic manipulation of SPA to combat obesity. These studies suggest that in orexin‐deficient conditions, reduced thermogenesis resulting from physical inactivity may lead to obesity. The orexin neurons functionally interact with DRN serotonin neurons to promote physical activity and energy expenditure while reducing energy intake, which well‐positions the orexin–serotonin system to integrate thermogenesis and vigilance state control. Empirical evidence shows that DRN serotonin is critical for motor control, and pharmacological manipulation of the serotonin system can be used as a therapeutic strategy to enhance energy expenditure, and to treat weight gain. The role of serotonin in the control of feeding and energy balance is complex, as use of antidepressants such as SSRIs result in obesity (Kivimaki et al., 2010). These findings are in accordance with the metabolic syndrome observed in SERT deficient mice (X. Chen et al., 2012; Yabut et al., 2019; Zha et al., 2017). Several SSRIs influence other neurotransmitter pathways (Bhagwagar et al., 2004; Chau et al., 2011; Fink & Gothert, 2007; Raeder et al., 2006; Zhou et al., 2005) and weight gain is not consistently observed following SSRI therapy (Hainer et al., 2006; Schwartz et al., 2004; Serretti & Mandelli, 2010). As mentioned above, future studies should focus on selective serotonin and orexin agonists, and serotonin antagonists, for development of therapeutic interventions against obesity. The orexin–serotonin system senses metabolic signals, and activates structures such as the motor cortex to modify behavioral outputs based on energy needs. Furthering our understanding of neural computations and anatomical and functional interactions among brain structures will not only provide us with a more comprehensive view of the functions of the orexin–serotonergic system, but also help in the development of new treatments for various types of diseases including metabolic, movement, and mental health disorders.

## CONFLICT OF INTEREST

The authors have declared no conflicts of interest for this article.

## AUTHOR CONTRIBUTIONS

**Vijayakumar Mavanji:** Writing – original draft (lead); writing – review and editing (equal). **Brianna Pomonis:** Resources (supporting); writing – original draft (supporting); writing – review and editing (supporting). **Catherine Kotz:** Conceptualization (lead); funding acquisition (lead); project administration (lead); supervision (lead); validation (lead); writing – original draft (equal); writing – review and editing (lead).

## RELATED WIREs ARTICLES

Central dopaminergic circuitry controlling food intake and reward: Implications for the regulation of obesity

## Data Availability

Data sharing is not applicable to this article as no new data were created or analyzed in this study.
